# Optimizing the Geriatric Mental Health Workforce Through Innovative Approaches

**DOI:** 10.1093/geroni/igab046.106

**Published:** 2021-12-17

**Authors:** Ana Jessica Alfaro, Rachel Rodriguez, Michele Karel

## Abstract

The drastic demand for geriatrics-trained providers in medical and mental healthcare persists years after the Institute of Medicine first highlighted this need (2008; 2012). New innovative approaches must instead optimize the current workforce through leveraging existing geriatric experts’ knowledge and skills related to working aging adults. This symposium will highlight four approaches spanning post-licensure education to using technology to deliver specialized services and training. First, Dr. Gregg will discuss the evaluation of an advanced topics workshop in Geropsychology which has significantly enhanced depth of Geropsychology competencies for psychologists working in primarily rural areas. Next, Dr. Asghar-Ali will describe the multi-modal interactive geriatric educational opportunities for interprofessional staff developed by the South East Texas Geriatric Workforce Enhancement Program (SETx GWEP). He will discuss how these training opportunities have been tailored to address the impact of COVID-19 and healthcare disparities among older adults. Third, Dr. Filips will present an evaluation of a consultation model in which a geriatric psychiatrist provides tele-consultation in a 5-state region to rural aging Veterans with complex medical and behavioral comorbidities. Finally, Dr. Beaudreau will describe adaptations to a national VA Problem Solving Training program for mental health clinicians of older Veterans with complex comorbidities. Dr. Karel, VA National Geriatric Mental Health Director, will serve as discussant and comment on the ways in which these novel approaches are meeting the ever-growing need for competent geriatric mental health providers.

